# Cellular architecture and transmitter phenotypes of neurons of the mouse median raphe region

**DOI:** 10.1007/s00429-016-1217-x

**Published:** 2016-04-04

**Authors:** Katalin E. Sos, Márton I. Mayer, Csaba Cserép, Flóra S. Takács, András Szőnyi, Tamás F. Freund, Gábor Nyiri

**Affiliations:** 1 Laboratory of Cerebral Cortex Research, Institute of Experimental Medicine, Hungarian Academy of Sciences, Budapest, 1083 Hungary; 2 János Szentágothai Doctoral School of Neurosciences, Semmelweis University, Budapest, 1085 Hungary

**Keywords:** Median raphe, Paramedian raphe, Stereology, Serotonin, 5-HT, ePET, PET-1, VGLUT3, VGAT, Immunohistochemistry

## Abstract

The median raphe region (MRR, which consist of MR and paramedian raphe regions) plays a crucial role in regulating cortical as well as subcortical network activity and behavior, while its malfunctioning may lead to disorders, such as schizophrenia, major depression, or anxiety. Mouse MRR neurons are classically identified on the basis of their serotonin (5-HT), vesicular glutamate transporter type 3 (VGLUT3), and gamma-aminobutyric acid (GABA) contents; however, the exact cellular composition of MRR regarding transmitter phenotypes is still unknown. Using an unbiased stereological method, we found that in the MR, 8.5 % of the neurons were 5-HT, 26 % were VGLUT3, and 12.8 % were 5-HT and VGLUT3 positive; whereas 37.2 % of the neurons were GABAergic, and 14.4 % were triple negative. In the whole MRR, 2.1 % of the neurons were 5-HT, 7 % were VGLUT3, and 3.6 % were 5-HT and VGLUT3 positive; whereas 61 % of the neurons were GABAergic. Surprisingly, 25.4 % of the neurons were triple negative and were only positive for the neuronal marker NeuN. PET-1/ePET-Cre transgenic mouse lines are widely used to specifically manipulate only 5-HT containing neurons. Interestingly, however, using the ePET-Cre transgenic mice, we found that far more VGLUT3 positive cells expressed ePET than 5-HT positive cells, and about 38 % of the ePET cells contained only VGLUT3, while more than 30 % of 5-HT cells were ePET negative. These data should facilitate the reinterpretation of PET-1/ePET related data in the literature and the identification of the functional role of a putatively new type of triple-negative neuron in the MRR.

## Introduction

The median raphe region (MRR) plays a fundamental role in regulating cortical network activity, as well as subcortical functions (Bohut 1997; Vertes et al. 1999). It participates in several aspects of fear behavior, its lesion disrupts the acquisition of conditioned fear memory, and it is associated with specific subtypes of anxiety disorders (Avanzi and Brandão 2001; Avanzi et al. 2003; Silva et al. 2004; Borelli et al. 2005; Dos Santos et al. 2005; Ohmura et al. 2014; Peters et al. 2014; Zangrossi and Graeff 2014). Its functional alterations may also lead to disorders, such as schizophrenia or major depression (Aghajanian and Marek 2000; Hensler 2006). However, its precise cellular composition with regard to transmitter phenotypes is still unknown.

The MRR is located in the midline of the brainstem and consists of the MR and the paramedian raphe (PMR) subregions. The MRR used to be known as a serotonergic nucleus; however, several studies have already reported the presence of non-serotonergic neurons (Gras et al. 2002; Jackson et al. 2009) as well. Four cell populations can be distinguished based on 5-HT, glutamate, and gamma-aminobutyric acid (GABA) contents: (1) some neurons contain only 5-HT (serotonin only, SO cells); (2) others show only vesicular glutamate transporter type 3 expression (VGLUT3 only, GO cells); (3) in the third group, both molecules are detectable (serotonin and VGLUT3, SG cells) (Fremeau et al. 2002; Shutoh et al. 2008); (4) while the fourth group of cells is GABAergic (Stamp and Semba 1995; Serrats et al. 2003; Calizo et al. 2011) and only contains vesicular GABA transporter (VGAT). No studies have investigated the possible occurrence of cells that would express none of these molecules or the ratios of these four cell types in the MRR.

Serotonergic and glutamatergic MRR cells project densely to several forebrain areas (Vertes et al. 1999; Azmitia 1978; Köhler 1982; Aznar et al. 2004; Varga et al. 2009). Serotonergic neurons desynchronize the hippocampal activity and disrupt rhythmic discharge of septal cholinergic and GABAergic neurons (Assaf and Miller 1978; Kinney et al. 1996; Vertes and Kocsis 1997). VGLUT3 containing glutamatergic neurons suppress the hippocampal ripple activity and disrupt memory consolidation (Wang et al. 2015), and both of these cells can innervate more forebrain areas simultaneously (Szőnyi et al. 2014). Although serotonergic and glutamatergic neurons are frequently investigated, it is unknown how many of them possess both transmitters.

Efforts to genetically identify and modify serotonergic cells led to the discovery of the E26 transformation-specific transcription factor PET-1 and its enhancer region ePET (Hendricks et al. 1999). The generation of PET-1-Cre and ePET-Cre transgenic mouse lines promised efficient manipulation of the 5-HT containing neurons and provided tools for numerous studies (Braz et al. 2009; Hodges et al. 2009; Hawthorne et al. 2010; Liu et al. 2010; Depuy et al. 2011, 2013; Spaethling et al. 2014; Wang et al. 2015).

In this study, we investigated the number of cells of different cell populations in the MRR. In the whole MRR, 2.1 % of the neurons were 5-HT, 7 % were VGLUT3, and 3.6 % were 5-HT and VGLUT3 positive; whereas 61 % of the neurons were GABAergic. Surprisingly, 25.4 % of the MRR neurons were only positive for the neuronal marker NeuN and were negative for 5-HT, VGLUT3, and VGAT, and because of several reasons detailed below, these cells are highly unlikely to be false negative. Furthermore, using one specific commonly used ePET-Cre transgenic mouse line (Jackson Laboratories), we show that more than half of the SO neurons are negative for ePET, and about 38 % of the ePET cells only contain VGLUT3. Therefore, ePET is not specific to serotonergic cells, since it is present in a portion of SO, GO, SG cells, and also in some of the unidentified neuronal population.

## Materials and methods

### Animals and perfusions

All experiments were performed in accordance with the Institutional Ethical Codex and the Hungarian Act of Animal Care and Experimentation guidelines, which are in concert with the European Communities Council Directive of September 22, 2010 (2010/63/EU). The Animal Care and Experimentation Committee of the Institute of Experimental Medicine of the Hungarian Academy of Sciences and the Animal Health and Food Control Station, Budapest, have also approved the experiments.

VGAT-IRES-Cre mice were crossed with Gt(ROSA)26Sor_CAG/ZsGreen1 or with Gt(ROSA)26Sor_CAG/tdTomato reporter mice, and ePET-IRES-Cre mice were crossed with Gt(ROSA)26Sor_CAG/ZsGreen1 mice (Jackson Laboratories). Their offspring showed genetically coded specific fluorescent labeling in VGAT or ePET expressing neurons. Scott et al. (2005) described in detail the construction of this ePET-Cre mouse line. Three 42 days old male VGAT-ZsGreen1 offspring mice (named GM1; GM2; and GM3), one 42 days old male VGAT-tdTomato mouse (named GM4), one 42 days old male C57BL/6J wild-type mouse (named WT), and three 49 days old male ePET-ZsGreen1 mice (named PM1; PM2; and PM3) were used. For perfusion, mice were anesthetized with isoflurane, followed by intraperitoneal injection of an anesthetic mixture (containing 8.3 mg/ml ketamine, 1.7 mg/ml xylazine-hydrochloride, and 0.8 mg/ml promethazinium-chloride) to achieve deep anesthesia. Then, mice were transcardially perfused first with PBS (0.9 % NaCl in 0.1 M phosphate buffer) solution for 2 min, followed by 4 % paraformaldehyde for 40 min, and finally with 0.1 M phosphate buffer (PB) for 10 min.

### Fluorescent immunohistochemistry

After perfusion of mice and removal of their brains from the skull, coronal sections were cut on a Leica VT1200S vibratome at 60 µm from the whole MRR and collected in stereological order into eight vials. This was followed by washing out the fixative in 0.1 M (PB) for 1 h. Then, the sections were cryoprotected sequentially in 10 % (overnight) and 30 % (2 h) sucrose in PB and freeze–thawed five times over liquid nitrogen. This was followed by several washing in 0.1 M PB (3 × 10 min) and tris-buffered saline solution (TBS, 3 × 10 min). Then, sections were incubated in TBS containing 1 % human serum albumin, 0.1 % Triton™ X-100, and 700 μg/ml Digitonin (all from Sigma-Aldrich) for 1 h to achieve better penetration.

Vials were separated into two groups (even and odd sections) that were used for experiment type A and B, respectively (Table 2). Even-numbered sections were used to demonstrate the ratios between the 5-HT and/or VGLUT3 positive neurons, while odd-numbered sections were used for determining the number of other neurons.

### Antibodies

Table 1 summarizes information about the primary and secondary antibodies. Previously, we extensively tested the rabbit anti-VGLUT3 antibody in experiments using VGLUT3 knock out animals (Szőnyi et al. 2014). Immunostaining of VGLUT3 in raphe neurons was also tested by others (Mintz and Scott 2006; Shutoh et al. 2008). The mouse anti-NeuN antibody labels a neuron specific DNA binding nuclear protein and is widely used to identify neurons (Mullen et al. 1992). To increase the accuracy of the measurements, we counted only the DAPI stained nuclei of cells. The rat and rabbit anti-5-HT antibodies were characterized before (Amilhon et al. 2010; Fox and Deneris 2012), they labeled the same cells, and also labeled the expected population of neurons, which further confirm their specificity. In the experiment, where the expression pattern of ePET was analyzed, we found a mismatch between ePET and 5-HT expression. These surprising results prompted us to test whether it is possible that some cells only express the synthesizing enzyme of serotonin, but the enzyme does not produce detectable levels of serotonin. Therefore, we tested whether all cells that are positive for the 5-HT synthesizing enzyme, tryptophan hydroxylase (TPH), are also positive for 5-HT. We colocalized rabbit anti-5-HT and mouse anti-TPH labeling (Fig. 2). We found that all 160 examined TPH labeled cells were also positive for 5-HT; consequently, 5-HT was always detected in TPH expressing cells. This shows that the sensitivity of 5-HT labeling cannot be responsible for the lack of labeling in some cells, because cells that express its synthesizing enzyme, TPH, always express detectable levels of 5-HT as well.

**Table 1:** Antibody specifications

Raised against	Raised in	Protein cc. of stock solution	Dilution	Source	Catalog number	Lot number	Characterized
VGLUT3	Rabbit	1 μg/μl	1:500	Synaptic Systems	135203		Szonyi et al. (2014)
NeuN	Mouse	1 mg/ml	1:500	Chemicon	MAB 377	LV 1359479	Mullen et al. (1992)
Serotonin	Rat	Not available	1:500	Merck Millipore	MAB 352	2168248	Amilhon et al. (2010)
Serotonin	Rabbit	25 μg/ml	1:10000	ImmunoStar	20080	1431001	Fox and Deneris (2012)
TPH	Mouse		1:3000	Sigma-Aldrich	T0678		Calizo et al. (2011)

Conjugated with	Raised in	Raised against	Dilution	Source	Catalog number	Molecule	
Alexa 594	Donkey	Mouse	1:500	Life technologies	A-21203	Full IgG	
Alexa 647	Chicken	Rat	1:500	Life technologies	A-21472	Full IgG	
Alexa 647	Donkey	Rabbit	1:500	Jackson immuno-research	711-605-152	Full IgG	
Cy3	Donkey	Rabbit	1:500	Jackson immuno-research	711-165-152	Full IgG	
Alexa 488	Donkey	Mouse	1:500	Life technologies	A21206	Full IgG	
Alexa 488	Donkey	Rabbit	1:500	Life technologies	A 21202	Full IgG	
DAPI	–	–	1:10.000	Sigma-Aldrich	D9564	–	

The antibody penetration into 60 µm-thick sections was examined rigorously using confocal imaging, and was found to be perfect even in the middle of the section. Secondary antibodies were extensively tested for possible cross-reactivity with other primary or secondary antibodies, but no cross-reactivity was found.

### Confocal microscopy

Image stacks were recorded by using a Nikon A1R confocal laser-scanning system built on a Ti-E inverted microscope with 0.45 NA CFI Super Plan Fluor ELWD 20XC Nikon objective and operated by NIS-Elements AR 4.3 software. Argon ion laser (457–514 nm, 40 mW), yellow DPSS laser (561 nm, 20 mW), violet diode laser (405 nm), and diode laser system (647 nm, 100 mW) were used as excitation lasers with appropriate filters. Images were acquired at a z-separation of 1 µm. Each section plane was identified by using the Mouse Brain Atlas (Paxinos and Franklin 2012).

### Stereology measurement

Unbiased design-based stereological measurements were carried out using the optical fractionator method (Sterio 1984; Gundersen 1986; West and Slomianka 1991; Schmitz and Hof 2005), which is based on the principle that one can accurately define the number of cells in the volume of interest by counting them in a predetermined fraction of the given volume (Dorph-Petersen et al. 2001). To get the total cell numbers, the number of counted cells is multiplied by the reciprocal of three different fractions: section, area, and thickness sampling fractions (West and Slomianka 1991). Using systematic random sampling in each experiment, every second section of the MRR was used; therefore, section sampling fraction was 0.5. In mounted sections, cells were counted only within a fraction of a predefined grid area. In the MR, this fraction was 15^2^/40^2^ µm in experiment type A and 15^2^/80^2^ µm in experiment type B. In the PMR, this fraction was 10^2^/80^2^ µm for both types of experiments. Finally, thickness sampling fraction was about 15/28 µm, because the average mounted section thickness was about 28 µm and counting performed only in a 15-µm-high counting cube. We used a guard zone of minimum 5 µm of tissue above and below the counting cube; however, for maximum accuracy, thickness sampling fractions were determined at every sampling site. Cells were counted inside the counting cubes or if they touched one of the inclusion planes of the counting cubes. Using these parameters, we directly identified the phenotype of about 13 % of the MR neurons and altogether counted about 12,300 nuclei in MRR in these animals. Cell counting was carried out in Stereo Investigator 10.0 stereology software (MBF Bioscience), while cells were identified parallel using NIS-Elements AR 4.2 software.

## Results

### Cell types of the MRR

**Fig. 1:**
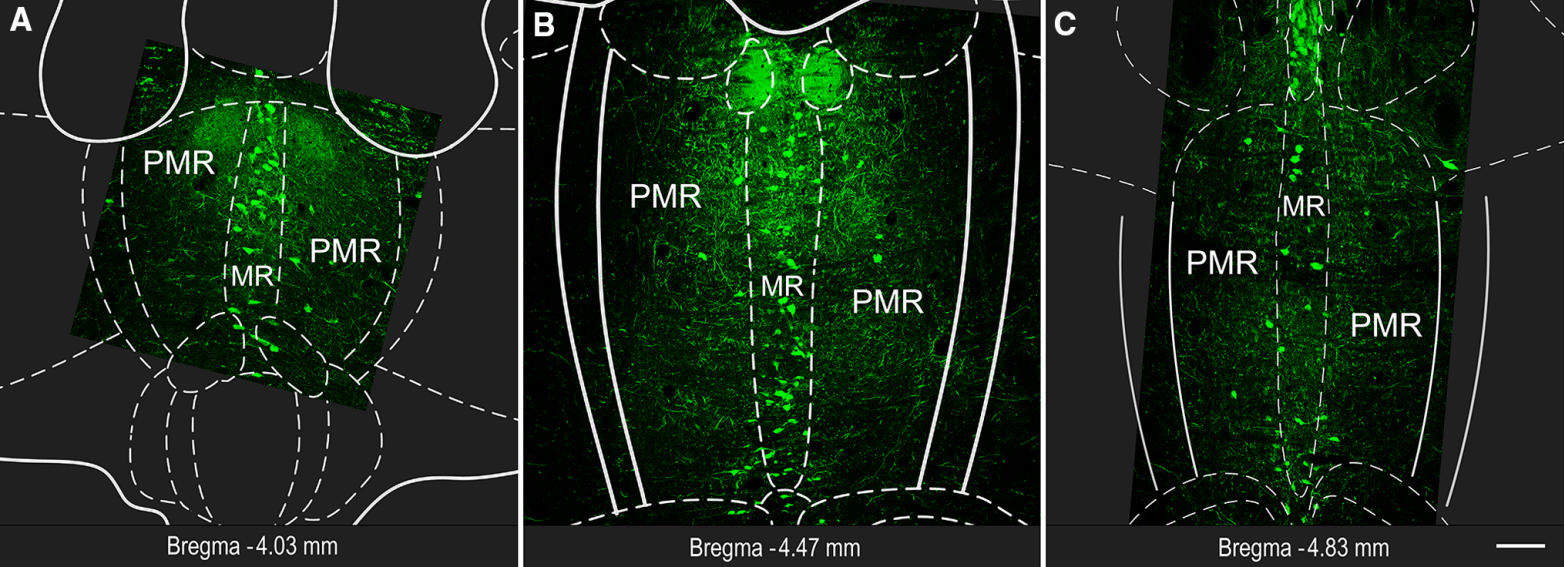
Fluorescent micrographs show representative MRR sections with 5-HT labeling. Subregions (MR and PMR) are defined based on the Mouse Brain Atlas (Paxinos and Franklin 2012). Position of the coronal section is indicated in each image, relative to the Bregma. *Scale bar* 100 µm for all images

**Fig. 2:**
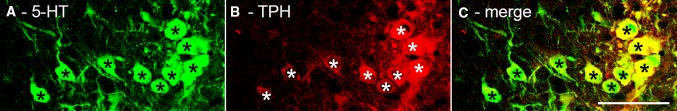
Confocal laser scanning images (**a**–**c**) show 100 % colocalization between the labeling of 5-HT and its synthesizing enzyme, TPH, in representative MR neurons (*asterisk*). *Scale* *bar* 50 µm for all images

**Fig. 3:**
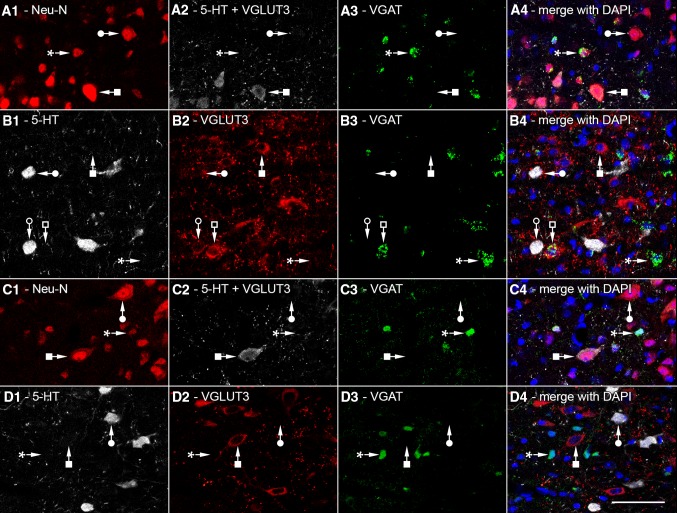
Confocal laser scanning images used for stereological measurement of different cell types of MRR in VGAT-IRES-Cre-ZsGreen (**a**, **b**) and VGAT-IRES-Cre-tdTomato mice (**c**, **d**). In both strains, genetically determined fluorescent markers labeling VGAT are shown in *green* in **a3**–**d3**. Nuclei were always labeled with DAPI (*blue*). In “experiment type B” (**a1**–**a4**; **c1**–**c4**), neurons were identified with Neu-N staining (*red*), while 5-HT and/or VGLUT3 positive cells were visualized in the same fluorescent channel (*white*). Some cells were only NeuN positive (*filled circles*), and some were positive for 5-HT and/or VGLUT3 (*filled square*); while some of them only VGAT positive (*asterisk*). In “experiment type A” (**b1**–**b4**; **d1**–**d4**), 5-HT positive (*white*) and VGLUT3 positive (*red*) cells were visualized separately. SO cells (*filled circle*), GO cells (*filled square*), SG cells (*empty circle*), VGLUT3 and VGAT positive cells (*empty square*), and only VGAT-positive cells (*asterisk*) could be differentiated. *Scale bar* 50 µm for all images

**Fig. 4:**
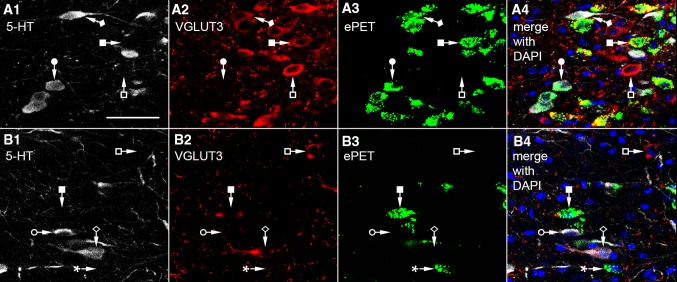
Confocal laser scanning images used for stereological measurement of different cell types of MRR in ePET-IRES-Cre-ZsGreen mice (**a**, **b**) in “experiment type A”. ePET labeling is *green*, nuclear DAPI labeling is *blue*, while 5-HT (*white*) and VGLUT3 (*red*) positive cells were visualized separately. Some SO cells express ePET (*filled circle*), while some are ePET negative (*empty circle*). Some GO cells were ePET positive (*filled square*), while some are ePET negative (*empty square*). Most SG cells are ePET positive (*filled diamond*), but some are ePET negative (*empty diamond*). Some cells expressed only ePET without any other labeling (*asterisk*). *Scale bar* 50 µm for all images

Using immunohistochemistry combined with stereological methods, we identified ten different types of neuronal phenotypes in the MRR. We used three kinds of genetically modified mouse strains and one wild-type mouse. We carried out two types of experiments, because we could use a maximum of four different fluorescent channels per experiment. In experiment type A, we focused on the identification of SO, GO, SG, VGAT, or ePET positive cells, while in experiment type B, we primarily focused on NeuN positive neurons that were negative for all other labeling (see Table 2). To label 5-HT, VGLUT3, and NeuN, we used immunohistochemistry; to stain the nuclei, we performed DAPI histochemistry and we used genetically expressed fluorescent markers for the visualization of VGAT and ePET. Using an unbiased stereological method, the combination of different mice and two types of experiments allowed the estimation of the absolute number of different cells in the MRR. The general labeling pattern of neuronal markers distributed in the MRR as expected, and neuronal markers could be clearly distinguished (Figs. 1, 2, 3, 4). We found that the genetic background did not have any effect on the estimated cell numbers.

**Table 2:** Experiment types, primary and secondary antibody combinations, and animals from different mouse strains

Mice (genetic background)	Experiment type A (here 5-HT and VGLUT3 cells could be distinguished, using different fluorescent channels)	Experiment type B (here 5-HT and VGLUT3 cells could not be distinguished, but other neurons could be detected in a separate channel with NeuN)
GM1, GM2, GM3 (strain: *VGAT*-*IRES*-*Cre*-*ZsGreen*) PM1, PM2, PM3—tested only with type A (strain: *ePet*-*ZsGreen*) WT—tested only with type A (strain*: C57BL/6J*)	Rat anti-5-HT/Alexa 647 Rabbit anti-VGLUT3/Cy3 DAPI	Rat anti-5-HT/Alexa 647 Rabbit anti-VGLUT3/A647 Mouse anti-NeuN/Alexa 594 DAPI
GM4 (strain: *VGAT*-*IRES*-*Cre*-*tdTomato*)	Rat anti-5-HT/Alexa 647 Rabbit anti-VGLUT3/Alexa 488 DAPI	Rat anti-5-HT/Alexa 647 Rabbit anti-VGLUT3/Alexa647 Mouse anti-NeuN/Alexa 488 DAPI

In all mouse strains, genetically determined fluorescent markers showed intensive expression in the soma of VGAT or ePET containing neurons (Figs. 3a3–d3, 4a3, b3). Some neurons expressed only these genetically determined markers: these are only VGAT positive (Fig. 3a3, c3, asterisk) or only ePET positive cells (Fig. 4b3, asterisk). In experiment type B, to determine the total number of neurons, we used Neu-N staining (Fig. 3a1, c1), which labeled all neuronal nuclei and with a lower intensity also the cytoplasm. Some cells were only NeuN positive, which were marked with filled circle in Fig. 3. In experimental type B, 5-HT and/or VGLUT3 positive cells were visualized in the same fluorescent channel (Fig. 3a2, c2). Some cells were positive for 5-HT and/or VGLUT3 (filled square in Fig. 3), while some were only NeuN positive or VGAT positive.

In experiment type A, 5-HT positive (Figs. 3b1, d1, 4a1, b1) and VGLUT3 positive (Figs. 3b2, d2, 4a2, b2) cells were visualized separately. Both of the markers showed intensive labeling in the somata and axon terminals. In the case of VGAT-Cre animals (Fig. 3), SO cells (filled circle), GO cells (filled square), SG cells (empty circle), VGLUT3 and VGAT positive cells (empty square), and VGAT-positive (GABAergic) cells could be differentiated. In the case of ePET-Cre animals (Fig. 4), some SO cells expressed ePET (filled circle), while some were ePET negative (empty circle). Some GO cells were ePET positive (filled square), while some were ePET negative (empty square). Most SG cells were ePET positive (filled diamond), but some were ePET negative (empty diamond). In both types of experiment, we performed DAPI staining, which labelled the nuclei in the blue fluorescent channel.

**Fig. 5:**
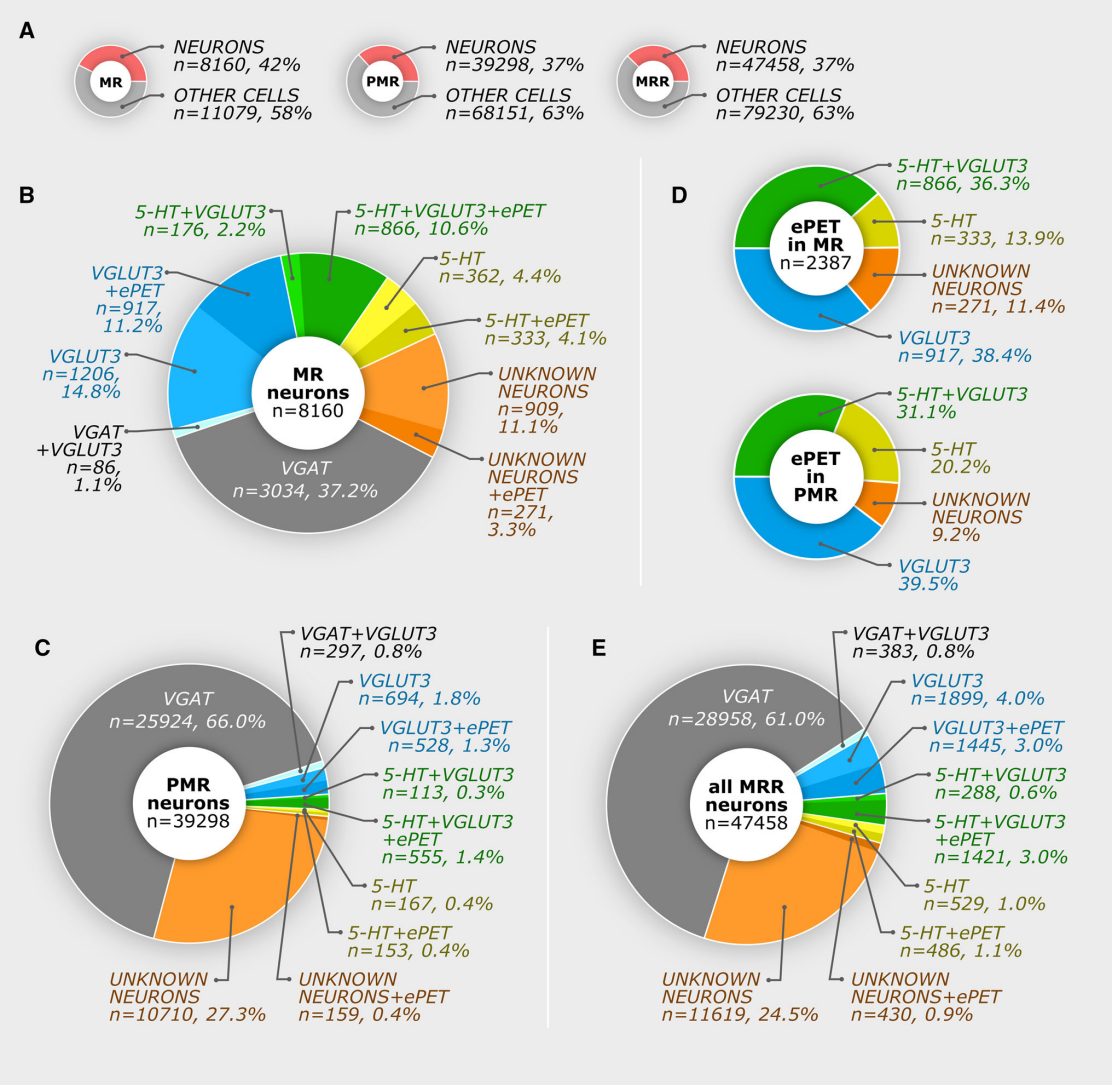
Number of cells and ratios in the median raphe region. Using original data presented in Tables 3, 4, these pie charts show calculated results originating from eight mice. **a** Ratios between neurons and non-neuronal cells in the MR, PMR, and MRR. **b**, **c** Different neuron types and ePET expression in the MR and PMR, respectively. **d** Distribution of 5-HT and VGLUT3 contents in ePET expressing neurons in the MR and PMR. Ratios of PMR neurons were defined with a semiquantitative method; therefore, the number of cells is not shown, but could be calculated from the other pie charts. **e** Summary pie chart about the whole MRR neurons

Stereological results are shown in Table 3 for MR and in Table 4 for PMR and the whole MRR. Based on these data, we calculated the ratios of the different cells types and plotted all results in Fig. 5. Briefly, in the MR, we found that only about 8.5 % of the neurons are SO cells, 26 % are GO cells, and about 12.8 % are SG double positive cells; whereas 37.2 % of the neurons are GABAergic, and 14.4 % of all MR neurons were triple negative for 5-HT, VGLUT3, and VGAT. In the PMR, we found that only 6.4 % of all neurons expressed 5-HT and/or VGLUT3, but these cells appeared only very close to the MR border, which was defined according to the Mouse Brain Atlas (Paxinos and Franklin 2012). 66.8 % of all PMR neurons expressed VGAT, while 27.7 % of the neurons were triple negative for 5-HT, VGLUT3, and VGAT. We never found that colocalization between 5-HT and VGAT and colocalization between VGLUT3 and VGAT occurred only very rarely (see Tables 3, 4; Fig. 5 for details).

**Table 3:** Number of cells in median raphe

The number of cells in median raphe in eight mice									
Labelled cell types	GM1	GM2	GM3	GM4	WT	PM1	PM2	PM3	Mean
Non-neuronal cells, only DAPI positive	11269	10449	10672	11928	–	–	–	–	11079
All neurons (calculated, using all mice, from the mean number of all labelled neurons)									8160
All 5-HT positive cells, without VGLUT3 labeling—abbreviated as “SO”	1310	515	573	662	640	807	351	697	695
And from these, ePET positive cells are:	–	–	–	–	–	380	240	378	333
All VGLUT3 positive cells, without 5-HT labeling—abbreviated as “GO”	1840	2078	2209	2055	2041	2209	2591	1957	2123
And from these, ePET positive cells are:	–	–	–	–	–	906	886	959	917
All 5-HT and VGLUT3 double positive cells—abbreviated as “SG”	627	890	904	940	1408	1427	1014	1126	1042
And from these, ePET positive cells are:	–	–	–	–	–	1002	705	891	866
All 5-HT and/or VGLUT3positive cells	3655	3450	3426	3620	4089	4862	3957	3780	3855
All VGAT positive cells, without 5-HT and VGLUT3 labeling	3105	3190	2619	3222	–	–	–	–	3034
All VGAT and VGLUT3 double positive cells, without 5-HT labeling	35	149	93	65	–	–	–	–	86
All NeuN positive cells (triple negative), without 5-HT, VGLUT3 and VGAT labeling	597	712	1632	1782	–	–	–	–	910
All ePET positive cells, without 5-HT and/or VGLUT3 labeling	–	–	–	–	–	68	451	296	271

**Table 4:** Number of cells in paramedian raphe and median raphe region

The number of cells in paramedian raphe						The number of cells in MRR (sum of the means of MR and PMR cells)
Labelled cell types	GM1	GM2	GM3	GM4	Mean	
Non-neuronal cells, only DAPI positive	69924	59634	71573	71472	68151	79230
All neurons (calculated, using all mice, from the mean number of all labelled neurons)					39298	47458
All 5-HT positive cells, without VGLUT3 labeling—abbreviated as “SO”	264	0	795	221	320	1015
And from these, ePET positive cells (calculated based on the proportions in Fig. 5b) are:					153	486
All VGLUT3 positive cells, without 5-HT labeling—abbreviated as “GO”	751	1143	2094	899	1222	3344
And from these, ePET positive cells (calculated based on the proportions in Fig. 5b) are:					528	1445
All 5-HT and VGLUT3 double positive cells—abbreviated as “SG”	1438	217	555	460	668	1710
And from these, ePET positive cells (calculated based on the proportions in Fig. 5b) are:					555	1421
All 5-HT and/or VGLUT3positive cells	2514	1535	3013	1785	2212	6067
All VGAT positive cells, without 5-HT and VGLUT3 labeling	33486	25728	25035	19445	25924	28958
All VGAT and VGLUT3 double positive cells, without 5-HT labeling	244	578	234	130	297	383
All NeuN positive cells(triple-negative cells), without 5-HT, VGLUT3 and VGAT labeling	9017	8646	11991	13820	10710	12049
All ePET positive cells, without 5-HT and/or VGLUT3 labeling (calculated based on the proportions in Fig. 5b)					159	430

The mouse MRR is a relatively small area. Dendritic trees of cells in MR or PMR cross their putative borders as well, making specific activation or deactivation of these putative subregions even more difficult. In addition, separate manipulation of the MR and PMR with optogenetic, electrical, and/or pharmacological tools would currently be a considerable challenge, if possible at all. Although genetic tagging can be used to selectively target a subpopulation of neurons, available methods would still manipulate cell in both MR and PMR together. Therefore, separation of MR and PMR is impossible even with optogenetic methods. Therefore, we calculated the number of cells for the whole MRR as well. We found that 13.5 % of the MRR neurons contained 5-HT and/or VGLUT3, while 61.8 % expressed VGAT, and 25.4 % belongs to the unidentified cell type (see Tables 3, 4; Fig. 5 for details).

### Distribution of ePET positive cells in the MRR

Previously, the PET-1 enhancer region, ePET, was thought to be expressed exclusively in serotonergic cells. However, we found a mismatch between ePET and 5-HT expression in MRR, as shown in Figs. 4, 5 and Tables 3, 4. We also found triple-negative NeuN positive neurons that were labeled with ePET. Although, in this experimental design, colocalization between the genetic markers (ePET and VGAT) is not possible, it is highly unlikely that these markers would colocalize, because ePET positive cells are mostly localized in MR, and most GABAergic cells are in the PMR. In addition, SO and SG cells that are partly ePET positive were never GABAergic. Furthermore, generally, excitatory and inhibitory neurons derive from different cell lines; therefore, it is highly unlikely that ePET would be localized in GABAergic cells as well. Therefore, we classified triple-negative and ePET positive cells as VGAT negative. Results clearly show that ePET is partially expressed in four different populations of neurons (Table 3; Fig. 5).

Because the “border” between MR and PMR is artificial, we have all reason to believe that all SO cells in MRR belong to the same neuronal population, regardless of their localization in MR or PMR, and the same is true for all other cell types. Therefore, the ratio of ePET positive cells in the PMR was calculated based on the ratios found in MR. However, to further confirm that ePET positive cells indeed comprise the same cell types at a similar distribution in the PMR, we defined the phenotype of the counted ePET positive cells in the PMR in experimental type A of one mouse. In that case, we used a semiquantitative approach that allowed the testing of colocalization of other markers with ePET positive cell on a larger number of cells. We collected ePET positive cells in PMR from a representative series of sections (a sample size was 425 ePET+ cells) in experimental type A. This was more optimal in this case, because ePET positive cells are rare in the PMR, and testing with the fully quantitative method would have provided an unacceptably small sample. The result is shown in Fig. 5d lower pie chart. This proportion is very similar to the ratio in the MR (Fig. 5d upper pie chart) that further confirms that these regions contain the same type of cells.

## Discussion

Using an unbiased stereological method, first, we estimated the average numbers of different cell types of the mouse MR and PMR areas. Second, we found that about a quarter of the neurons are negative for 5-HT, VGLUT3, and VGAT, which indicates the existence of a so far unrecognized cell population. Third, we found that ePET is not specific for 5-HT, because it is not present in all SO neurons, and it is expressed in GO and triple-negative neurons as well.

### Ratios of cell types in MR and PMR

MRR is a widely investigated brain area, and several physiological experimental manipulations (excitation, inhibition or lesions) target the whole MRR. The physiological role of individual cell populations could be studied using optogenetic manipulation of cells with certain neurochemical phenotypes; however, even this method cannot be cell type specific, because MRR neurons form neurochemically overlapping cell populations that hamper their separate investigation. Although intersectional genetics might help to separate these cells in the future, it is important to know their ratios; otherwise, even intersectional genetic experiments may be misinterpreted. In addition, our results can help to define the absolute number of cells that are modulated in optogenetic experiments.

5-HT and VGLUT3 neurons are abundant in the center of the MRR, and they are getting gradually less and less abundant outward from the center, and the steepness of the decrease in abundance is variable between mice. Therefore, there is no way to unbiasedly draw a border between MR/PMR based on staining pattern. Dendritic trees of cells in MR or PMR cross their putative borders as well making specific activation or deactivation of these putative subregions even more difficult, if possible at all. The mouse MRR is relatively small; therefore, even from the practical point of view, the separation of the MR and PMR may also seems to be less useful, because most types of experimental interventions would affect both MR and PMR in mice. These subregions contain the same types of cells, and during experiments using optical fibers the lateral and the conical light spread is about 500–1000 µm from the optical fiber tip (Adamantidis et al. 2007; Yizhar et al. 2011), which would affect the whole volume of median raphe region; therefore, at least in mice, data for the whole MRR seem to be more useful. However, because of historical reasons and also to allow a certain level of comparison with other species, we analyzed MR and PMR separately as well, for which the only unbiased and reproducible method was to use the stereotaxic Mouse Brain Atlas as reference (Paxinos and Franklin 2012).

Interestingly, based on these criteria, more than a third of the 5-HT and/or VGLUT3 containing neurons are in the PMR—mostly closer to the MR border. We found that about half of the serotonergic cells contained VGLUT3, whereas a third of the VGLUT3 neurons were positive for 5-HT as well. It is important to note that, while the MRR contains about 47,500 neurons, only about 13 % of them are investigated regularly, because these 5-HT and/or VGLUT3 positive cells are known to project to forebrain areas.

One-third of MR neurons and two-thirds of PMR neurons are GABAergic, and these cells never contained 5-HT. Although it may seem insignificant, we found that about 0.8 % of MRR neurons showed VGAT and VGLUT3 double positivity. This type of colocalization is also known in a population of hippocampal GABAergic interneuron (Somogyi et al. 2004), but their connections and role in the MRR is unknown.

### The quarter of MRR neurons belong to an unknown population

We found a previously unidentified cell population, which constituted more than 25 % of the MRR neurons. This group was triple negative for 5-HT, VGLUT3, and VGAT labeling, and was positive only for the neuronal NeuN staining.

These cells are highly unlikely to be false negative, because: (1) they were just as numerous close to the section surface, where no antibody penetration problems occur, (2) they were surrounded by other strongly positive cells, also indicating appropriate antibody penetration; furthermore, (3) these triple-negative cells were distributed homogeneously in the tissue, which means that it is not due to regional penetration differences. In addition, (4) about 28 % of neurons belong to this triple-negative population also in the PMR, where SO, GO, and SG cells are rare, so they are highly unlikely to be false negative of those populations. (5) We proved that 5-HT can indeed label all serotonergic cells, because all TPH positive cells were 5-HT positive. (6) Genetic labeling was always very strong, and the specificity of genetically labeled VGAT expression was extensively tested (Madisen et al. 1993; Vong et al. 2011).

This triple-negative cell type was completely overlooked in the literature, because only SO, GO, SG, and GABAergic neurons were investigated, and to the best of our knowledge, these markers have never been investigated in the same experiment. Triple-negative cells are unlikely to be glutamatergic, because other vesicular glutamate transporter (VGLUT1 or VGLUT2) positive cells have not yet been shown in the MRR (Hioki et al. 2010). Dopaminergic neurons of negligible density were observed in the rat MRR (Jahanshahi et al. 2013), and although its colocalization with 5-HT or VGLUT3 was not tested, triple-negative cells are much more abundant, therefore, highly unlikely to be dopaminergic. In the MRR, most neurokinin-1 receptor labeling did not colocalize with VGLUT3 (Commons and Serock 2009), but other neurons of the MRR were not tested. Somatostatin and galanin positive neurons were also observed in the MRR (Araneda et al. 1999), but their localization in VGLUT3 and/or all 5-HT positive cells was not tested. To the best of our knowledge, so far, no other types of neurons were identified in the MRR that could account for the amount of this new triple-negative type of neuron.

### Possible neurotransmitter phenotype plasticity of 5-HT and/or VGLUT3 containing neurons

Neurotransmitter phenotype plasticity has already been described in several areas of the mammalian brain (Baudry et al. 2010; Dulcis et al. 2013). For instance, during the normal development (between P15 and P90), dopamine neurons lose their VGLUT2 contents completely in ventral tegmental area and substantia nigra (Bérubé-Carrière et al. 2009). In the medial nucleus of the trapezoid body, GABAergic neurons transiently release glutamate, and then switch to glycine as a primary neurotransmitter (Demarque and Spitzer 2012). Glutamatergic granule cells of the dentate gyrus are initially GABAergic, and then express a dual GABAergic/glutamatergic phenotype before becoming purely glutamatergic, but they can transiently express a GABAergic phenotype when a state of hyperexcitability is induced in the adult rat (Gutiérrez et al. 2003). Interestingly, in the MRR in this study, the number of 5-HT and/or VGLUT3 containing neurons shows high variability among mice. Although their ages were similar, 5-HT could be detected in more cells in mouse GM1 compared with GM2; on the other hand, GM2 had more VGLUT3 positive GO and GS cells. However, the total numbers of 5-HT and/or VGLUT3 containing neurons are fairly stable among animals, which suggest that in some mice, serotonergic/glutamatergic ratios would change without changing the total number of main forebrain projection neurons: SO, GO, and SG cells. These shifts in ratios are unlikely to be due to technical reasons, because we used all sections of the MRR and collected a large systematic random sample; therefore, uneven or topographic cell distributions could not account for this variability. We may hypothesize that these SO, GO, and SG cells belong to the same neuronal population, which can change its transmitter phenotype, as a function of activity in MRR inputs. Other data also support this hypothesis. First, ePET was expressed in all these three types of neurons, and we found some triple-negative cells that were also labeled with ePET, which may represent either a transitional cell population or a developmental side-branch. Second, VGLUT3 accelerates 5-HT transmission at specific 5-HT terminals; its deletion decreased 5-HT tone in projection areas and decreased serotonin autoreceptor-mediated transmission in raphe, further suggesting a close cooperation of these transmitter systems in the same cells (Amilhon et al. 2010). Third, a robust change in serotonin content of raphe neurons has already been detected between postnatal day 22 and 61 (Rind et al. 2000). Our mice (P42, P49) had slightly different housing environments; some of them had many littermates, and others had less that may have caused a variation in regulation of their MRR.

### Expression of ePET is not restricted to serotonergic neurons

A large body of the literature, partly summarized by Deneris (2011), is based on the assumption that PET-1/ePET can faithfully identify serotonergic neurons. PET-1 was identified as a key factor that triggers the appearance of serotonergic phenotype (Hendricks et al. 1999). PET-1 was thought to play a role in the 5-HT neuron development and is required for anxiety-like and aggressive behavior (Hendricks et al. 2003). Based on those results, ePET (an enhancer region upstream of mouse PET-1-transcribed sequences) was identified and was thought to be a reliable marker for serotonergic cells (Scott et al. 2005).

Using a commonly used ePET-Cre transgenic mouse line that was described by Scott et al. (2005) and is now supplied by Jackson Laboratories; here, we studied the expression of ePET in different types of MRR neurons. Surprisingly, ePET is expressed not only in SO cells, but in GO and SG neurons as well. More than half of SO cells showed a lack of ePET expression, while almost half of GO neurons were also ePET positive. More than a tenth of all ePET expressing neurons were negative for both 5-HT and VGLUT3 labeling. In fact, a previous study has already also shown that 5-HT positive neurons are present in the raphe even in PET-1 knock-out mice, and they were estimated to be about 20–30 % of the 5-HT neuron population in wild-type mice (Kiyasova et al. 2011). PET-1-independent and dependent serotonergic cells have been shown to project to different brain areas (Kiyasova et al. 2011). Furthermore, non-serotonergic neurons were also found to be positive for PET-1 in several raphe nuclei (Pelosi et al. 2014).

Our results can be used to estimate the number and ratios of modulated neurons in previous and future studies, employing ePET/PET-1 transgenic mice, and facilitate the reinterpretations of data in the literature.
